# Financial Time Series Prediction Using Spiking Neural Networks

**DOI:** 10.1371/journal.pone.0103656

**Published:** 2014-08-29

**Authors:** David Reid, Abir Jaafar Hussain, Hissam Tawfik

**Affiliations:** 1 Department of Mathematics and Computer Science, Liverpool Hope University, Liverpool, United Kingdom; 2 School of Computing and Mathematical Sciences Liverpool John Moores University Liverpool, United Kingdom; Tel Aviv University, Israel

## Abstract

In this paper a novel application of a particular type of spiking neural network, a Polychronous Spiking Network, was used for financial time series prediction. It is argued that the inherent temporal capabilities of this type of network are suited to non-stationary data such as this. The performance of the spiking neural network was benchmarked against three systems: two “traditional”, rate-encoded, neural networks; a Multi-Layer Perceptron neural network and a Dynamic Ridge Polynomial neural network, and a standard Linear Predictor Coefficients model. For this comparison three non-stationary and noisy time series were used: IBM stock data; US/Euro exchange rate data, and the price of Brent crude oil. The experiments demonstrated favourable prediction results for the Spiking Neural Network in terms of Annualised Return and prediction error for 5-Step ahead predictions. These results were also supported by other relevant metrics such as Maximum Drawdown and Signal-To-Noise ratio. This work demonstrated the applicability of the Polychronous Spiking Network to financial data forecasting and this in turn indicates the potential of using such networks over traditional systems in difficult to manage non-stationary environments.

## Introduction

Most financial data is non-stationary by default, this means that the statistical properties, such as the mean and variance, of the data changes over time. These changes are a result of various business and economic cycles such as the high demand for air travel in the summer months effecting on exchange rates and fuel prices [1]. While isolated information is usually taken into account, for example in the current closing price of a stock, share or exchange rate, the consequences of this knock-on effect means that the long term study of the behaviour of a specific variable is not always the best indicator of future market behaviour.

Stock market fluctuations are a result of complex interactions and the effect of these fluctuations are often interpreted as a sequence of stock price time series plots [2]. The most significant variations that are looked for are: trend, periodic variations and day-to-day variations. Trend is an identifiable long term variation in the stock market time series, while the periodic variations follow either seasonal patterns or the business cycle in the economy. Short-term and day-to-day variations usually appear at random and are difficult to predict with the exception of the case of “special events” such as public holidays, specific product launch dates or predicable breaking news, but these are often the source for stock trading gains and losses, especially in the case of day traders [2], [3], [4].

The prediction of financial time series is notoriously difficult and a nontrivial problem since it depends not only on known but also on unknown (and often unknowable) factors and frequently data that is used for the prediction is noisy, uncertain and incomplete. The financial series are affected by many highly correlated economic, political and even psychological factors; as a result it has been suggested that some financial time series are not at all predictable [5], [6], [7]. Despite this, the practical prediction of financial time series attracts interest due to its potential for massive financial gain.

Researchers and practitioners have long been striving for an explanation of the movement of financial time series. In order to maximise profits from the liquidity market different forecasting techniques have been used by traders [8]. Assisted by computer technologies, traders no longer rely on a single technique to provide information about the future of the market. From purely statistical to esoteric methods of artificial intelligence, there are many choices of techniques which can be used to make a forecast. The traditional methods for financial time series forecasting are based around statistical approaches, none of which have proved to be completely satisfactory due to the nonlinear nature of most of the financial time series [9]. Other more advanced techniques have been used such as Support Vector Machine [10], genetic algorithms [11], [12], [13] fuzzy logic [14], have only achieved limited success and tend to be focused toward a particular application domain. Other methods that have been used for time series analysis also include detrended fluctuation analysis and detrended cross correlation analysis [15], [16]

Neural networks are believed to have great potential in the financial time series prediction domain due to their predictive ability, adaptability to different domains and robust behavioural characteristics in uncertain environments.

Multi-Layer Perceptrons (MLPs) have been successfully applied to a broad class of financial markets predictions [3], [8], [17], [18]. However, MLPs use computationally intensive and training algorithms such as error back propagation [19] and can easily get stuck in local minima. In addition, these networks have poor interpolation performance, especially when using limited training sets.

For some tasks, including time-series prediction, higher order combinations of some of the neural networks inputs or activations may be appropriate to help form good representations for modeling non-linear problems.

Higher-Order Neural Networks (HONNs) distinguish themselves from ordinary feedforward networks by the presence of high order terms in the network [20], [21], [22]. In the specific case of financial data predictions various forms of Higher Order neural Networks, including Pi-Sigma networks, Ridge Polynomial Networks, and Functional Link Networks have been applied; these show favourable results when compared to the performance of Multi-Layer Perceptron networks [22], [23], [24] in such complex environments.

Traditional neural networks use firing rate as a way to encode and process information. This is normally done by averaging the weighted sum of inputs and mapping this onto a continuous activation function. This destroys important information that may be encoded into the patterns of activation such as strong outlying correlations in specific patterns of data.

It has been observed [25] that correlations and interdependencies exist between markets so that some stocks show a large degree of coupling. It has also been suggested that changes to highly coupled stocks could help predict agitation in financial markets. Yet this dynamic is very difficult to encode into traditional neural networks. The Index Cohesive Force; or ICF is the ratio between the raw and residual stock correlations [26]. This correlation may be used by neural networks tuned to react to individual correlations in the market; that is by neural networks that encode information temporally and that explicitly preserve the relationship between potentially useful correlations.

More recently, Spiking Neural Networks (SNNs) have demonstrated the power of this type of neural networks in solving difficult problems in complex and informationally “messy” environments.

Unlike the older class of neural networks, this so called “third generation” of neural networks use spike times to encode data [27], [28]. It is argued that these new generations of neural networks are potentially much more powerful at predicting non stationary patterns, and are, in reality, a superset of the “traditional” or rate encoded neural networks hither to used [29].

SNNs are inherently suited to manage highly non-linear and temporal based input data that traditional neural networks struggle with. A small number of researchers have applied spiking neural network systems in order to classify and predict financial time series data [30], [31]. However, most spiking neural networks applied to financial forecasting, if not direct adaptations of traditional neural networks, can indirectly trace their origins to older types of neural networks already applied to this field. Typically such networks involve choosing a particular feature set which is then used to explicitly analyse and classify financial data. Apart from Glackin [32], who uses fuzzy logic to analyse patterns of spike trains in a SNN as the basis for financial forecasting in a novel way, in most SNNs applied to this area of financial forecasting often the temporal financial information gets overwhelmed by other factors or is just simply ignored.

As such, despite their potential, the application of SNNs into meaningful financial data predictions seems to be under-explored; which in turn suggests that this particular field of research is very much in its infancy.

Therefore, it is proposed in this paper that the explicit engineering of the temporal aspects of the financial data into a spiking neural network make the SNNs more suited to time series analysis than traditional rate encoded neural networks that preceded them or the SNN derived from thee rate encoded networks [33], [34], [35].

The remainder of this paper is organized as follows: section 2 discusses the methodology and algorithmic design choices for the proposed Spiking neural network. Section 3 describes the proposed Polychronous neural network paradigm for financial time series prediction. Simulation results and discussions are presented in sections 4 and 5 respectively. Section 6 covers conclusions and the future research directions.

## Methods

### Spiking Neural Networks for Financial Data Forecasting

Temporal point processes are often used to describe signals that occur at a finite set of time points. Unlike continuous valued processes, which can take on countless values at each point of time, a temporal point process use binary events that occur in continuous time [36], [37]. As most signal processing techniques are designed for continuous valued data, care needs to be taken to transfer the probability theory of point processing [38] into meaningful spike data. This can be done in three ways; by encoding the information by spike counting, by relative spike timing or by absolute spike timing [39].

The SNN proposed in this paper uses absolute spike times to directly correlate with the closing prices of several financial markets. This is an appropriate correlation for a number of reasons:

1. markets prices are measured at discrete time intervals,
2. markets have a discrete closing and opening price, and
3. markets are always “naturally” bounded by ultimately finite resources and are often far more restricted than this by being “bounded rationally” [40].

SNNs used so far in financial modeling and predication use a simple Leakey Integrate and Fire model. This paper postulates the argument that it is logical to use a neuron model that has the capability for significant temporal control in order to accurately model the temporal nature of the financial time series. For this reason the Izhikevich neural model [41], [42] was favoured over the LIF model [29].

Izhikevich argues [41] that a potentially major contributing factor to learning is often neglected in spiking neural network research; that of axonal delay. Consequently our system acknowledges this by using the Spike Time Dependent Plasticity (STDP) learning rule [43], [44], which is derived from traditional Hebbian learning.

This gives our SNN the capability to rapidly recognize complex patterns, often with a single neuron's spiking output being a flagged as a result of a pattern of other afferent neurons' reaction to stimulation.

This idea is referred to as the theory of neuronal group selection (TNGS) or neural Darwinism [45], [46]. It is this novel method that the authors of this paper utilise in order to predict values in an evolving and non-stationary financial environment.

### Experimental Design

When comparing two fundamentally different types of neural networks and a standard linear classifier system a number of compromises have to be taken. While it may be possible to perform a direct comparison between SNN, traditional NNs, and linear systems; this often necessitates modification of the SNN to such an extent that it resembles the function in a similar way to a standard Linear Predictor Coefficients model or traditional rate encoded neural network. By modifying the SNN in this way it may lose many of its advantageous characteristics.

The experiments performed for this paper therefore did not adopt the aforementioned approach and instead use the SNN firing patterns in order to select candidate routes that match the real world financial data. In order to make an equivalent traditional neural network or a linear classifier performs in this way would necessitate very many runs of the network requiring feedback of potential solutions into the network over a very long time period. Even if these modification where made to the different types of network, their fundamental intrinsic differences would still mean that a direct comparison may not be achievable.

Care had to be taken in the design of the systems involved in this experiment as design factors can have a major impact on the accuracy of network forecast; for example: the selection of the input-output variables; the choice of data, the initial weight state, and the stopping criterion during the training phase can influence results. Similarly issues such as the learning parameters, the number of nodes and the activation function are also important. The way the data is pre-processed may also have a significant effect. In order to accurately diagnose the mechanisms working in a system, it is important to present the data to each of the systems in as “pure” and unmodified way as possible, which minimises pre-processing and promotes simplicity.

In this research work, three financial time series signals are considered as shown in Table 1.

**Table 1:** Time series data used in the experiments.

Data Signal	Dates	Number of data points
Oil Price	01/01/1985 to 01/11/2008	389
IBM	17/05/1961 to 02/11/1962	360
US/Euro rate	03/01/2000 to 04/11/2005	1525

The IBM closing stock price was used, as it is a well-known time series described by Box [47]; the foreign exchange market is used as this is the largest and most liquid of the financial market with an estimated $1 trillion traded everyday [8], [48]; and the price of oil is used as this is becoming an important time series and it exhibits extreme non stationary behaviours. The financial data forecasting problem is reliant on predicting various prices, as in the case of forecasting return or log returns [49], [50], [51].

The oil and the exchange rates time series were obtained from the Federal Reserve banks and the Board of Governors, which was established by the Congress in 1913 and which is shown in the following website http://economagic.com/ecb.htm/fedstl.htm, while the IBM common stock closing price time series was taken from the Time Series Data Library [52].

**Figure 1:**
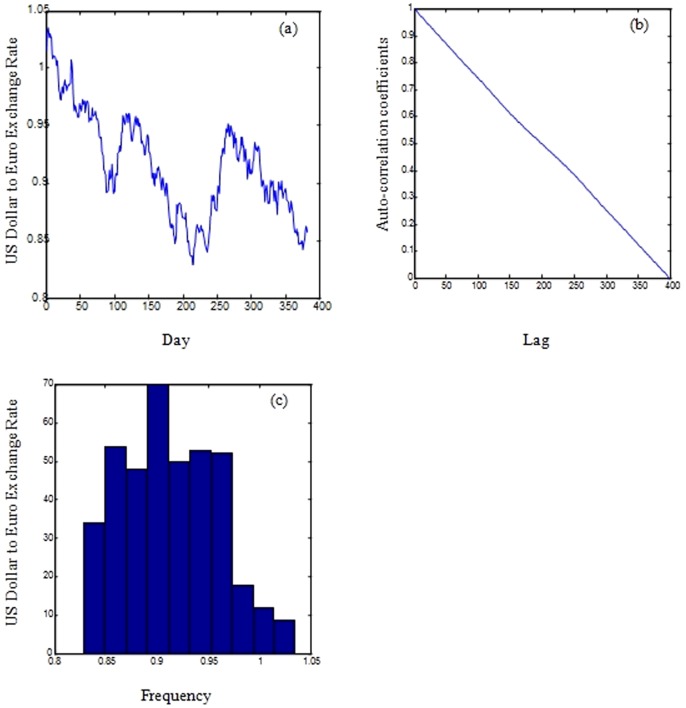
Exchange rate between the US Dollar and the Euro (a), the correlogram of the exchange rate between the US dollar and Euro signal (b), and histogram of the exchange rate between the US dollar and the Euro (c).

The exchange rate is an important economic measure in the international monetary market. Its importance comes from the fact that both governments and companies use it to make decisions on investment and trading. It is believed that the exchange rates have direct influence to all changes in the economic policies, and as a result, any attempt to predict the behaviour of an economy is materialised in the foreign exchange rates. The foreign exchange rates time series show high nonlinearity, very high levels of noise, and significant nonstationarity. In this paper the exchange rate between the US Dollar which is acting as a reference currency and the euro is considered as shown in Figure 1 (a).

**Figure 2:**
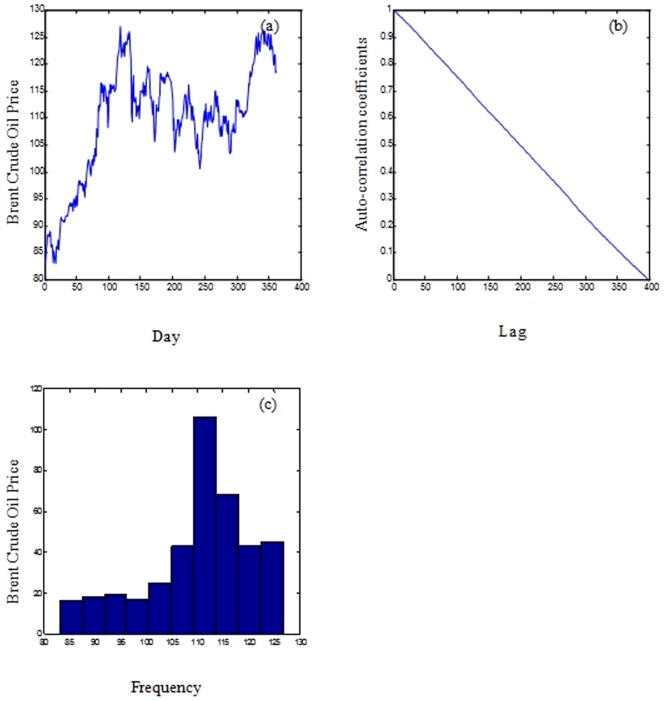
Brent crude oil price (a), the correlogram of the oil data signal (b), and histogram of the Brent crude oil price (c).

The Oil prices data is a monthly data that represents the Oil price of West Texas Intermediate crude and which covers the interval between 01/01/1985 and 01/11/2008 as shown in Figure 2 (a).

**Figure 3:**
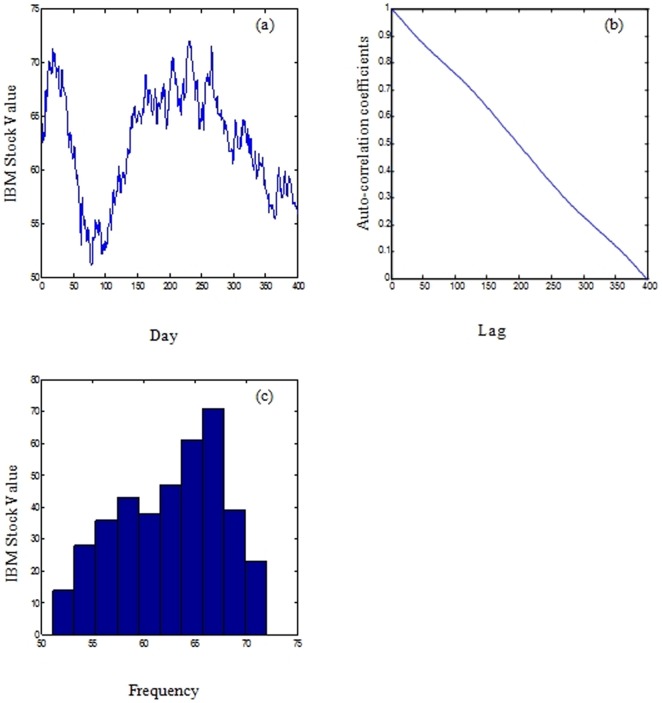
IBM stock values (a), the correlogram of the IBM stock value signal (b), and histogram of the IBM stock values (c).

On the other hand, Figure 3 (a) shows the IBM common stock price in the period between 17/05/1961 to 2/11/1962. The IBM closing price, owned by the world's largest information technology company was selected as it is a well-known time series, described by Box et.al [53].

As shown in Figures 1, 2, and 3 (b), the correlograms of the IBM, the oil and the daily exchange rate between the US Dollar and the Euro time series indicated that the autocorrelation coefficient drops to zero for large values of the lag. As a result, we can conclude that the time-series are non-stationary signals. Furthermore, the signals exhibit high volatility, complexity, and noise as shown in the histogram images (refer to Figures 1, 2, and 3(c))

The data is scaled to accommodate the limits of the network's transfer function. Manipulation of the data using this process produces a new bounded dataset. The calculation for the standard minimum and maximum normalization method is as follows:
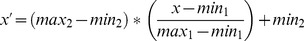
(1)where *x*′ refers to the normalized value, x refers to the observation value (original value), min_1_ and max_1_ are the respective minimum and maximum values of all observations, and min_2_ and max_2_ refer to the desired minimum and maximum of the new scaled series.

The statistical measures used in evaluating the performance of the neural networks are the Mean Squared Error (MSE), the Normalized Mean Squared Error (NMSE), and the Mean Absolute Error (MAE). However, for financial time series forecasting, the aim of the prediction is also to achieve trading profits based on prediction results in addition to the forecasting accuracy. As a result, financial criteria were used as the primary test as to whether the model is of economic value in practice [8].

The prediction performance of this work were measured using three financial metrics, and two statistical and signal processing metrics, as shown in Table 2.

**Table 2:** Signal processing and trading simulation performance measures.

Metrics	Calculations
**Annualized Return (AR)**	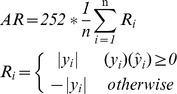
**Maximum Drawdown (MD)**	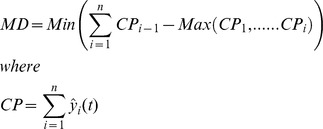
**Signal to Noise Ratio (SNR)**	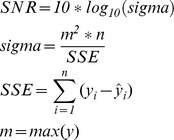
**Normalized Mean Square Error (NMSE)**	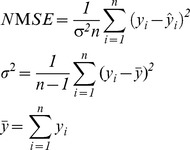
**Annualized Volatility (AV)**	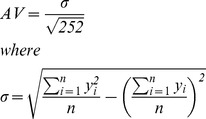

n is the total number of data patterns.

*y* and 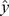 represent the actual and predicted output value.

The objective of using financial metrics is to use the networks predictions to ultimately generate profit, whereas the statistical and signal processing metrics were used to provide accurate tracking of the signals, for forecasting accuracy purposes.

**Figure 4:**
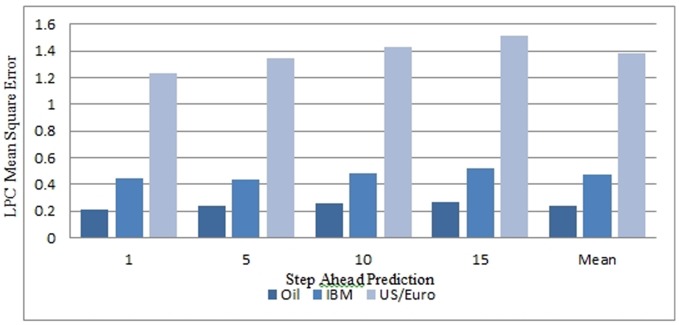
Step Ahead Prediction Mean Squared Error (MSE) for Linear Predictor Coefficient Model.

**Figure 5:**
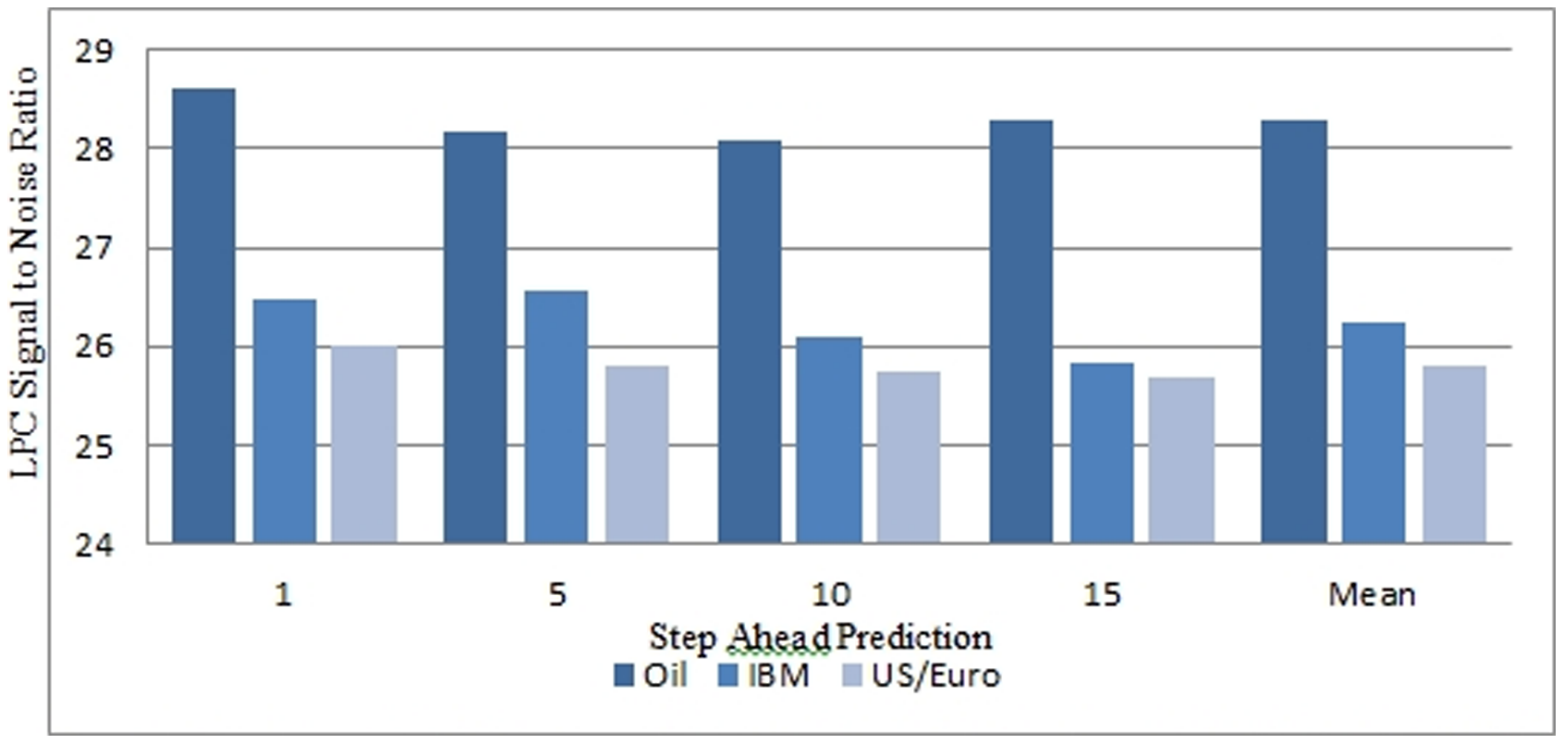
Step Ahead Prediction Signal to Noise Ratio (SNR) for Linear Predictor Coefficient Model.

Choosing a suitable forecasting horizon is a very important step in financial forecasting. From the trading aspect, the forecasting horizon should be sufficiently long such that excessive transaction cost resulting from over-trading is avoided [10]. Similarly the forecasting horizon should be short enough as the persistence of financial time series is of limited duration. Thomason in his work [54] suggested that a forecasting horizon of five days is a suitable choice for daily data. To systematically select the appropriate prediction horizon, linear predictor was utilised for the prediction of 1-step, 5 steps, 10 steps, and 15 steps prediction for the three time series and evaluated using the SNR and the MSE performances. The simulation results as shown in Tables 3 and 4 and in Figures 4 and 5 indicated that no significant performance changes using the MSE and the SNR were noticed for 5 step ahead prediction.

**Table 3:** 1,5,10 and 15 step ahead prediction Mean Squared Error for the Linear Predictor Classifier.

Step Ahead Prediction	Oil Price	IBM Stock Value	US/Euro Exchange Rate
1	0.2075	0.4471	1.2342
2	0.2411	0.4335	1.3486
10	0.2591	0.4802	1.425
15	0.2658	0.5169	1.5107
Mean MSE	0.243375	0.469425	1.379625

**Table 4:** 1,5,10 and 15 step ahead prediction Signal to Noise Ratio for the Linear Predictor Classifier.

Step Ahead Prediction	Oil Price	IBM Stock Value	US/Euro Exchange Rate
1	28.6086	26.4725	25.9932
2	28.1832	26.5651	25.7979
10	28.0913	26.0978	25.7307
15	28.3049	25.8201	25.6745
Mean SNR	28.297	26.23888	25.79908

Hence, considering the trading and prediction aspects from both literatures and the simulation results, this research work consequently implements a 5-days steps ahead forecasting horizon.

### A Polychronous Spiking Neural Model for Financial Time Series Prediction

Given the aforementioned considerations, we propose using Polychronous Spiking Neural (PSN) network. Recently, Johnson and Venayagamoorthy have shown how real values can be encoded into such a network [55]. However their work focuses on encoding non temporal data into such a network whereas our focus is on encoding temporal financial data into such a network. Financial data usually has temporal ordering and precise timing as major factors contributing to different patterns of market behaviour.

Several possible encoding methods were considered, especially inter spike interval representing market values at particular points in time; encoding financial data as a neuronal gray value bit pattern, however it was decided to map the values onto distinct neurons. The reasons for adopting this methodology are:

1. Simplicity, it is relatively easy to scale and then to map the financial data onto a set of neurons;
2. Easy to decode, the nature of the Polychronous Spiking Neural network means that at any time interval many different patterns of neuronal activation can exist in the network representing possible “candidate” solutions. If a complex encoding scheme is used this may hide, or even destroy, the causal chain of neuronal activity;
3. Easy to encode; temporal information is encoded directly into the network without manipulation;
4. Easy to interpret; causal neuronal chains in the network correspond to different candidate solutions; these in turn can be mapped back to real data.

Training of the network started with scaling and rounding the raw data to the nearest integer so that it would map onto 100 neurons. These neurons represent the real values of the data (in the case of US/EU exchange rate data this also required multiplication by 100 in order access the significant digits).

**Figure 6:**
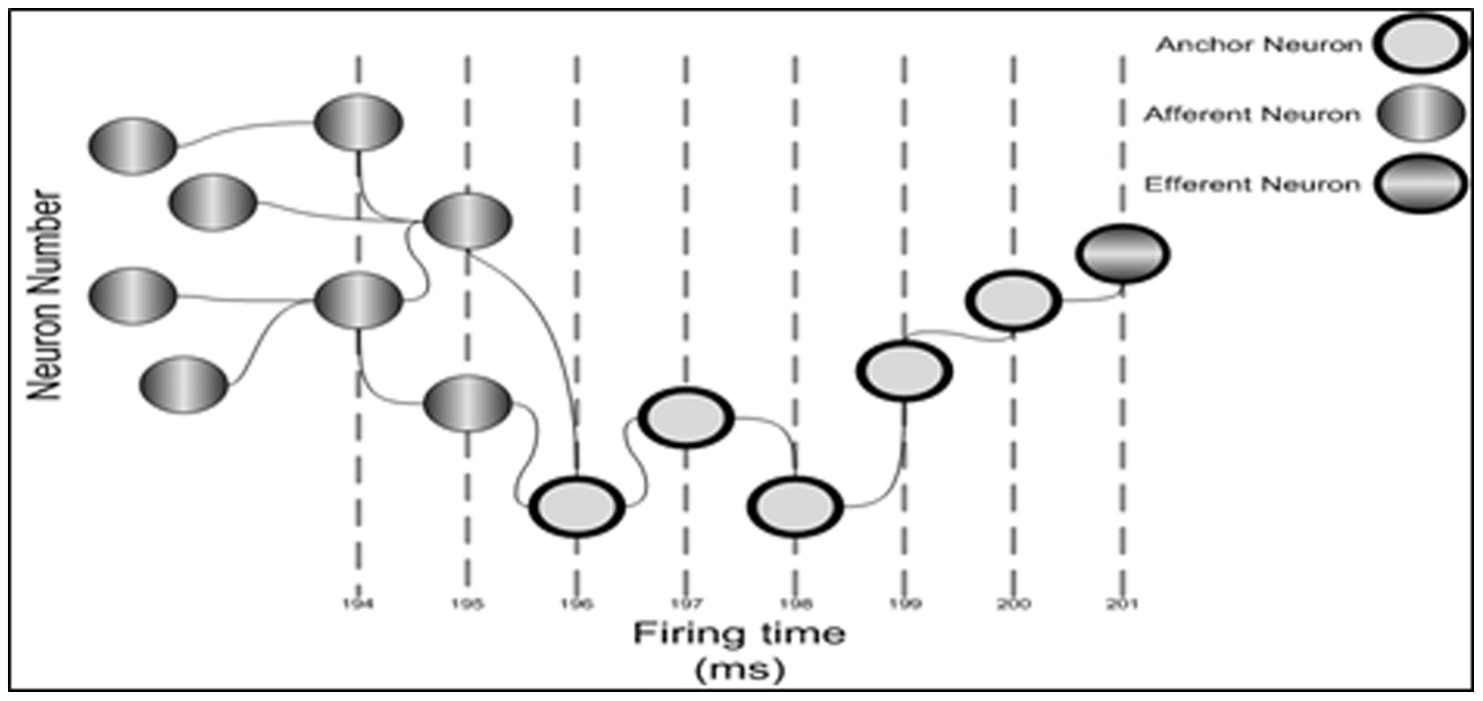
Activation chains of firing patterns through the network instigated by thalamic input signals representing real value data.

This scaled data was then presented to the network via “thalmic input”. This is represented by the value *W* in equation 2 and amounts to the total influx on spiking information at a particular time step after synaptic delays have been accounted for. It is this variable that we use to fire the relevant neuron represented the scaled financial data value. These firing patterns are shown in Figure 6.
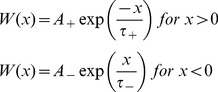
(2)where parameters A+ and A− are parameters dependant on the value of the current synaptic weight and *τ*_+_ and *τ*_−_ are time constants/boundaries normally in the order of 10 ms.

During experimentation three different training architectures were investigated:

1. Randomly connected neurons with random delays, these along with their afferent neurons were updated after each training cycle.
2. Bands of connected neurons (with a 10 neuron neighbourhood), with each band and their afferent neurons being updated at each training cycle.
3. A focused single neuron and its afferent neurons being updated with each training cycle.

**Figure 7:**
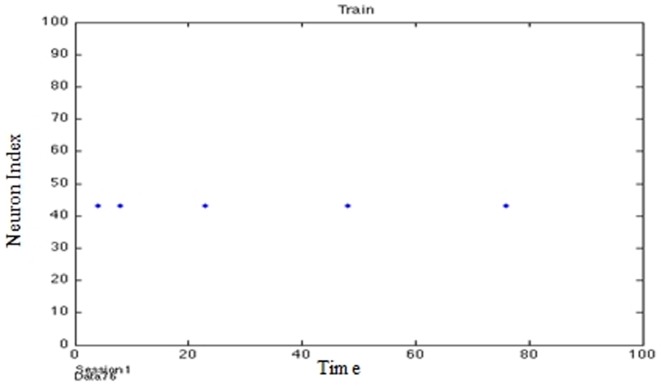
Training the PSN (no Gamma Cycle).

**Figure 8:**
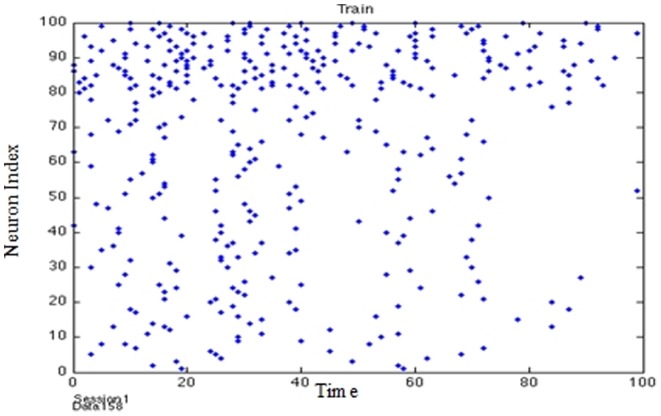
Training the PSN (Gamma Cycle).

It was found that this last method exhibited faster training by a factor of 4 and produced comparable results to the other 2 methods. It was noted that during training the system periodically entered bursts of activity indicated that afferent neurons were being activated (in a manner suggesting a gamma cycle) in a focused way (Figures 7 and 8).

Training consisted of presenting the financial data values 100 times to the network (experimentation with lower numbers of training session, down to 10, also produced similar results).

The 5 days up to the midpoint of the data (values 195–200) were then taken as the “anchor” neurons. These are neurons that have been influenced, or will influence, other significant neurons. The network then looked for all possible pre-synaptic firing patterns in the previous 200 afferent neuron values that were similar to the previous 200 real data values, with a tolerance of ±5 ms. These were labelled as “candidate” paths.

After running the network the candidate path that most closely resembled the real data was chosen and a prediction made based on the continuation of firing of the candidate path to the efferent neuron most likely to fire in the 201^st^ or 205^th^ spike time. This is illustrated in Figure 6.

Different combinations of possible neuronal paths were ranked according to when neurons fired and which afferent neurons influenced them. A path through the network that resembles the real financial data pattern was deemed to be the best approximate forecast of the current and, via subsequent firings, future market conditions.

## Simulation Results

**Figure 9:**
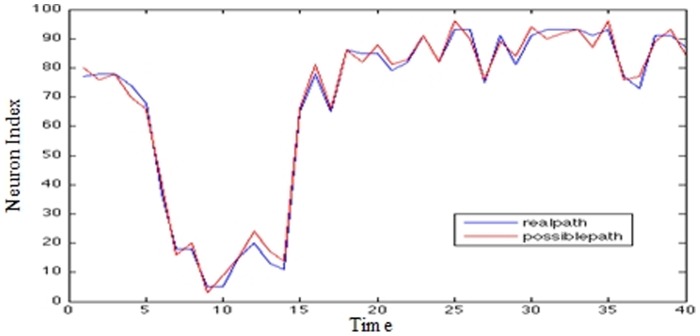
IBM stock prices (5-step prediction).

**Figure 10:**
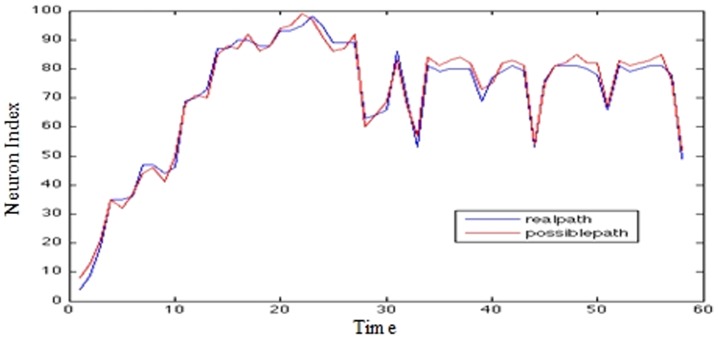
Brent crude oil prices (5-step prediction).

**Figure 11:**
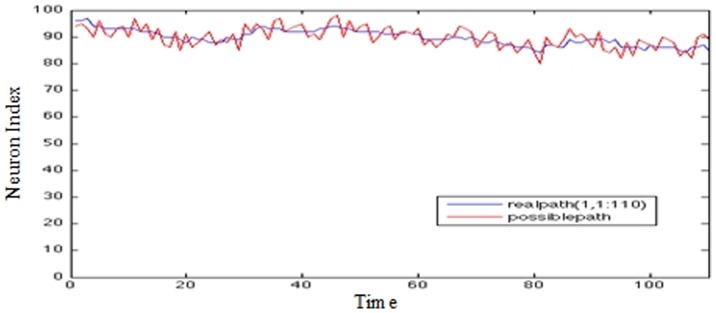
US/EU exchange rate (5-step prediction) - The Y axis represents the Neuron Index; The X axis represents time. In Figures 9–11 The ‘Blue’ line represents the real data over time; the red line represents the closest synfire chain; i.e. chain of ‘firing’ neurons.

Graphs presented in Figures 9, 10, and 11 show on the x-axis 400 trading days and on the y-axis the closing values representing the number of the maximally fired neuron. The data from day 0–200 forms the basis of the prediction. The movement traced in the Y axis represents the maximally fired neuron at a particular time (±5 ms), the error at a particular instance can be deduced by how far a neuron is from the real data value when it fired.

It can be seen that for the most part, the system has been trained to fire neurons at approximately the correct times in order to mimic the movement of the real data.

From this a 5-step prediction is made based on the firing chain of events pattern that occurred in the afferent neurons over the 0–200 time frame. These neurons are used to fire an efferent neuron on day 201 or on day 205; the neurons following the market behaviour are used to see what other neurons can be fired if they fire at the times specified.

Initially, we experimented with the Linear Predictor Coefficients (LPC) model [56] for the prediction of the 3 types of financial times serried shown in this paper. The simulation results indicated that the LPC models generate less favourable annualised return results, in comparison with our neural network models. This was found to be consistent with previous findings in this area. For examples, Ferreira [51] showed that MLP network obtained results better than a LPC model, for all financial time.

In Table 5, 5-step prediction results are shown for the closing prices for Brent crude oil, IBM stocks and US/Euro exchange rates, using three types of neural networks:

**Table 5:** 5-step time-series prediction results.

Measure	Network	Oil Price	IBM	US/Euro rate
AR	LPC	−19.4035	−5.5758	15.1846
	MLP	2.6385	1.6523	2.9824
	DRPNN	14.6108	−0.2958	8.63152
	PSN	94.5051	96.2261	27.162
SNR	LPC	28.1832	26.5651	25.7979
	MLP	15.06	7.87	16.21
	DRPNN	22.98	18.43	20.52
	PSN	30.1939	92.3077	81.6514
NMSE	LPC	0.2411	0.4335	1.3486
	MLP	3.3703	10.7787	1.1719
	DRPNN	0.6098	0.9437	0.4337
	PSN	0.0883	0.0662	14.5187
AV	LPC	180.2314	94.4259	1.0142
	MLP	17.7153	20.1867	10.8801
	DRPNN	17.6485	20.1777	10.8731
	PSN	55.8574	65.0968	20.9892

1. A Linear Predictor Coefficients (LPC) model (ARMA based)
2. A traditional MLP network,
3. A Dynamic Ridge Polynomial Neural Network DRPNN, a recurrent form of higher order neural networks which proved to perform favourably in the prediction of financial time series [24], and
4. Our proposed PSN network.

The performance of the PSN network is primarily evaluated using the signal processing and trading metrics defined in Table 2 in which the prediction performance of our networks was evaluated using three financial metrics, where the objective is to use the networks predictions to make financial gain, and two statistical metrics which are used to provide accurate tracking of the signals.

The ability of the networks was evaluated by the Annualized Return (AR), a real trading measurement which is used to test the possible monetary gains and to measure the overall profitability in a year (252 working days), through the use of the ‘buy’ and ‘sell’ signals [17]. The AR is often the most significant economic measurement for a specific market. This is a scaled calculation of the observed change in the time series value, where the sign of the change is correctly predicted.

The 5-step prediction results from Table 5 are, for the most part, consistent. For annualised return the PSN consistantly had the best results by a large margin. The DRPNN produced a better prediction compared to MLP and LPC for Oil price and US/Euro rate prediction but on the other hand generated a small loss in oil price prediction (−0.2958). Overall for this most important indacator, PSN exhibited highly favourable performance.

Maximum drawdown (MD) is an indicator of the risk of a particular portfolio. It measures the largest single drop from peak to bottom in the value of a portfolio, before a new peak is achieved. It is the percentage loss that a fund incurs from its peak net asset value to its lowest value. Taking into account scaling factors all three of the neural networks concur.

The Signal to Noise Ratio (SNR) compares the level of a desired signal to the level of background noise; in this case it is the ratio of useful information about a portfolio compared to false or irrelevant data. The 5-step predictions show consistent results. Again, the PSN has the best SNR; for IBM shares and the US/Euro rate this is particularly pronounced.

Normalised Mean Squared Error (NMSE) shows overall deviations between predicted and measured values. NMSE is a useful measure because if a system has a very low NMSE, then it indiates that it is correctly identifing patterns.The PSN produced significantly better NMSE results for the IBM and oil prices, consistantly achieving NMSE error values less than 1 (0.0883 and 0.0662 respectively), which are well below the NMSE values for the MLP, DRPNN and LPC predictors. DRPNN has the best performing neural network for the US/EU data having a NMSE of only 0.4337.

Annualized Volatility (AV) is the measure of the changeability in asset returns, which means less volatility is preferable. It describes the variability in a stock price and is used as an estimate of investment risk and for profit possibilities. The volatility is of great interest for financial analyst and provides useful information when estimating investment risk in real trading. This is calculated as the standard deviation of the portfolio price return over a working year (252 days). The PSN results obtained are consistent with the advantages that this network shows over the other systems tested.

In order to assess the statistical validity of the performance of the PSN network, a paired t-test [57] was conducted to determine if there is any significant difference among the proposed spiking neural network for financial time series prediction and the MLP and the DRPNN based on the absolute value of the error on out of sample data. The calculated t-value showed that for all the predicted signals the proposed Polychronous Spiking Networks technique outperform the other neural networks predictors with α = 5% significance level for a one tailed test. This is confirmed by the simulation results as shown in Table 6 for out of sample data.

**Table 6:** Mean absolute value of the error for out of sample data.

Neural Network Predictors	IBM dataset	Oil price	US/EU exchange rate
PSN	0.0321	0.0067	0.0151
DRPNN	1.4596	0.9809	0.5786
MLP	7.6383	8.7041	0.1220

We have utilised 50% of the data as out of sample for the T-test experiments. These results clearly indicate that the proposed spiking neural network is significantly better than the DRPNN and the MLP networks in predicting these financial time series datasets.

## Discussion

This work has aimed to demonstrate the applicability of a particular type of spiking neural network (the PSN) to financial forecasting in a non-stationary environment and shows that, given the right settings, it can function more effectively than both standard LPC system and traditional rate encoded neural networks.

As can be seen by the NMSE results in Table 5, the PSN developed for this research is dependent on good spread of values that can be mapped onto the network in an effective manner. If the mapping is well distributed the results are highly favourable, whereas if the distribution is poor the results are less favourable. In either case we have shown that the PSN can make a good prediction at 5-steps into the future. Statistical validation of the results of the out of sample results confirms the significance of the improved performance shown by our proposed network

It should also be noted that although the NMSE is poorer for the PSN for US/EU exchange rates than with some other data sets, the value achieved by the main measure of success (AR) is excellent (more than trebling the annual revenue achieved by the best results of the traditional neural networks; 27.162 compared to 8.63152 for DRPNN). This behaviour is not surprising as axonal delays are an important aspect of learning in the PSN. It is reasonable to assume that the ability to explore different paths through this network will directly influence learning. If a narrow spread of values is used then the network will have less opportunity to explore different solutions. This can be considered, using traditional rate encoded neural network terminology, to be equivalent to the PSN converging onto local minima [58]. However, it should be emphasised that unlike traditional neural networks, this behaviour has a less significant effect on the overall final prediction capability of the network. Our PSN exhibited faster training capability in that stable results were achieved after only 10 training cycles.

However, comparing training cycles between the different types of neural network needs to take into account that the PSN functions in a fundamentally different way to the other neural networks; unlike the other neural networks and the LPC, the PSN uses a number of different spiking signal patterns in each of its training cycles. These spike patterns effectively compete as to which pattern should persist to the next epoch. This raises one of the practical challenges with the current application of the PSN; this is in classifying and grading the very large number of candidate solutions generated. As the PSN uses spike trains and delays to influence Spike Timed Dependence Plasticity learning a very large number of candidate routes can be derived from a relatively small number of neurons. We used 100 neurons over 200 time steps. This has the potential to generate 1.866524e+160 possible different permutated routes through the network, each representing different forecasts. Given the very high number of candidate routes generated an exhaustive search would take a very long time. However, in practice poorly performing routes can be dismissed or are, as in our system, automatically discounted by the network as training takes place. This was done in our system by using simple Euclidean distance to exclude out poor routes.

One major concern for the prediction of financial time series is the fact that the published literature has mostly concentrated on the nonlinearity of the signals and ignored the non-stationary properties of the financial data due to the difficulties involved with the implementation of adaptive filtering [59]. This has led researchers to assume that predictability is only possible if a stationary relationship can be found between the present and past values of the signals [60]. However, Kim et al. [61] showed that financial data can be considered close to stationary if it varies slowly; they used the Korea stock price index as an example of a non-stationary signal that can be modelled as an asymptotic stationary auto-regressive AR process. As this condition does not apply to all types of financial data, this work also supports the argument that the utilisation of the PSN in financial time series prediction promises to offer a favourable alternative. The results of the experimentation performed in our research supports this hypothesis.

## Conclusions

We have applied a specific type of spiking neural network, a Polychronous Spiking Network (PSN) to solve non-stationary financial data prediction problems in order to exploit the temporal characteristics of the spiking neural model in an appropriate way.

Our spiking neural network model adopted the Izhikevich neural architecture using axonal delays encoding the information such that its temporal aspects were preserved.

Experiments using our PSN showed that it outperforms a standard Linear Predictor Coefficients (LPC) Model and more traditional, rate-encoded, neural networks, namely Multi-Layer Perceptrons (MLP) and a Dynamic Ridge Polynomial Neural Network (DRPNN), when solving three different financial datasets of IBM stock data, the US/Euro exchange rate and the price of Brent crude oil. The PSN superior performance was evidenced by its performance using the key financial measure of Annualised Return (AR) and the Mean Square Error for 5-step ahead prediction. Other metrics such as Maximum Drawdown, Signal-To-Noise ratio, and Mean Square Error were used, and supported in large the PSN's superior performance over the other systems.

This work has both demonstrated the applicability of a particular type of PSN to financial data forecasting and its potential to perform more effectively than traditional neural networks in non-stationary environments.

Future work will focus on the exploration of improved ways to map the data onto the PSN, and the adaptation of the classification and grading of candidate solutions for parallel architectures so that different parts of the problem can be solved by decompositions of the search space of candidate solutions.
